# Case Report: McLeod syndrome caused by a nonsense variant c.484C > T (p.Gln162Ter) in the *XK* gene in a Chinese Han family

**DOI:** 10.3389/fgene.2026.1939479

**Published:** 2026-10-01

**Authors:** Luochu Dong, Mange Liu, Ying Tan

**Affiliations:** 1 4 + 4 Medical Doctor Program, Chinese Academy of Medical Sciences and Peking Union Medical College, Beijing, China; 2 Department of Neurology, Peking Union Medical College Hospital, Beijing, China

**Keywords:** case report, hyperCKemia, hyperkinetic movements, McLeod syndrome, neuroacanthocytosis, *XK* c.484C > T variant

## Abstract

**Introduction:**

McLeod syndrome (MLS) is a rare X-linked neuroacanthocytosis caused by pathogenic variants in *XK*, and is characterized by hyperkinetic movements, peripheral neuropathy and myopathy with hyperCKemia, acanthocytosis and cardiomyopathy. Here, we report a mainland Chinese Han family with McLeod syndrome carrying the *XK* variant c.484C > T, which resulted in a premature stop codon at position 162 (p.Gln162Ter), which, to our knowledge, has not been reported in association with McLeod syndrome in Asian populations; notably, the variant is not listed in the ISBT online Kx allele table at the time of writing.

**Methods:**

Clinical, laboratory, imaging, electrophysiologic, immunohematologic, and genetic data were extracted from the proband’s medical records, with targeted evaluation of first-degree relatives. The variant was interpreted according to ACMG/AMP criteria; ColabFold v1.5.5 was used for *in silico* structural prediction using wild-type and mutant sequences. The case report was prepared in accordance with the CARE guidelines.

**Results:**

A 59-year-old man was admitted with an 8-year history of progressive gait disturbance and choreiform movements, later accompanied by cognitive decline and distal-predominant upper limb weakness. Examination showed generalized areflexia, markedly elevated creatine kinase, bilateral caudate head atrophy on brain MRI, and axonal sensorimotor polyneuropathy on nerve conduction velocity (NCV) testing, with chronic neurogenic changes on needle electromyography (EMG). Peripheral smear revealed acanthocytosis in more than 60% of red blood cells, and serologic testing demonstrated markedly reduced reactivity with anti-Kell (K) and anti-Cellano (k) antibodies, suggesting reduced or absent Kx antigen and supporting a McLeod phenotype; whole-exome sequencing identified a hemizygous *XK* variant c.484C>T in the proband, and his asymptomatic daughter was heterozygous. The diagnosis of McLeod syndrome was established, and supportive management was recommended; at telephone follow-up, hyperkinetic movements persisted and symptomatic medication had not yet been initiated because of concerns about adverse effects.

**Conclusion:**

This family broadens the ethnic and clinical spectrum associated with the *XK* c.484C > T (p.Gln162Ter) variant and highlights the value of integrating acanthocyte assessment, McLeod blood group phenotyping, and genetic sequencing in the evaluation of males with hyperkinetic movements, peripheral neuropathy, and hyperCKemia to enable earlier diagnosis of MLS.

## Introduction

McLeod syndrome (MLS), also termed XK disease, is one of the core neuroacanthocytosis (NA) syndromes. It is an ultra-rare X-linked multisystem disorder caused by pathogenic variants in *XK*, with an estimated prevalence of approximately 1–5 per 1,000,000 people (Jung et al., 2011; Jung et al., 2007; Jung et al., 2004). Hematologically, MLS is defined by absence of the Kx red blood cell antigen and markedly reduced Kell system antigens (the McLeod phenotype), which has important implications for transfusion practice (Jung et al., 2007; Jung et al., 2004). The other core NA syndrome is VPS13A disease (chorea-acanthocytosis, ChAc), which is similarly rare and shares many clinical manifestations with MLS, including hyperkinetic movement disorders, cognitive and psychiatric symptoms, peripheral neuropathy, myopathy, and multiorgan involvement (Jung et al., 2011; Jung et al., 2007; Jung et al., 2004; Walker et al., 2011). Despite this overlap, MLS typically has a later age at onset, less prominent orofacial dystonia and tongue biting, but more frequent and clinically significant cardiomyopathy and arrhythmias, which contribute substantially to morbidity and mortality (Jung et al., 2011; Jung et al., 2007; Jung et al., 2004).

Although the diagnosis of MLS can be definitively established by demonstrating the McLeod red cell phenotype and/or identifying a pathogenic *XK* variant (Jung et al., 2007; Jung et al., 2004; Walker et al., 2024), recognizing MLS as a diagnostic possibility in routine practice remains challenging because of marked inter-individual variability in clinical presentation. Thus, detailed reports of genetically confirmed cases remain important for defining the phenotypic spectrum and supporting *XK* variant interpretation (Jung et al., 2011; Jung et al., 2007; Jung et al., 2004; Walker et al., 2024).

Here, we report a mainland Chinese Han family with McLeod syndrome due to the *XK* nonsense variant c.484C > T (p.Gln162Ter). To our knowledge, this allele has been documented only once previously, in an African-American man with a different set of clinical features (Walker et al., 2024; ISBT, 2021). By integrating multimodal assessments of the proband with targeted evaluation of first-degree relatives, we aim to further delineate the phenotypic spectrum of MLS (Jung et al., 2011; Jung et al., 2007; Jung et al., 2004; Walker et al., 2024).

## Methods

This is a case report with family investigations conducted at a tertiary care hospital. Clinical information, including detailed neurological examinations, laboratory data, neuroimaging, electrophysiology, immunohematologic and genetic data was obtained from the electronic medical record. Whole-exome sequencing of the proband was carried out in a certified clinical genetics laboratory; the identified *XK* variant was confirmed in the proband and tested in first-degree relatives by Sanger sequencing. In silico protein structure prediction was performed using ColabFold v1.5.5 (AlphaFold2 using MMseqs2) with the wild-type and truncated XK amino-acid sequences (Mirdita et al., 2022). Variant interpretation followed the ACMG/AMP criteria (Richards et al., 2015). Peripheral blood smears were prepared after mixing the blood sample 1:1 with isotonic saline, and were examined under light microscopy. Kell blood group typing was performed using a conventional tube agglutination method to assess Kell (K) and Cellano (k) status. The case report was compiled in line with the CARE guidelines for case reports, and written informed consent for publication was obtained from the participants in the study (Gagnier et al., 2014).

### Case description

A 59-year-old man presented to our hospital with an 8-year history of progressive gait instability and hyperkinetic movements. In 2017, without any apparent precipitating factor, he gradually developed an unsteady gait and hyperkinetic movements characterized by hand flinging, head shaking, sighing, tugging at his sleeves, and scratching. No perioral or periocular movements were reported, and these symptoms gradually increased in frequency. In 2023, he developed weakness in extension of the left index finger, accompanied by memory decline and word-finding difficulty. In 2024, left-hand muscle atrophy became apparent, and brain MRI performed at a local hospital showed bilateral caudate head atrophy. In 2025, he developed numbness of the right upper and lower limbs, most pronounced in the right hand, followed by right-hand weakness and muscle atrophy. In August 2025, he presented to our hospital with serum creatine kinase of 3,655 U/L (24–195 U/L). The patient’s hyperkinetic movements and unsteady gait were recorded in November 2025 during his inpatient diagnostic workup at our center (Supplementary Video S1).

His medical history included poorly controlled type 2 diabetes mellitus and a prior left radical nephrectomy with benign pathology. He also had a right shoulder fracture treated with MRI-compatible internal fixation. He denied any history of blood transfusion, reported light smoking of approximately one pack per week for 15 years, and denied heavy alcohol use.

His family history was notable for a younger brother who died at the age of 50 from a condition described by the family as “muscle atrophy,” approximately 10 years after symptom onset. No other relatives were known to have similar manifestations (Figure 1).

**FIGURE 1:**
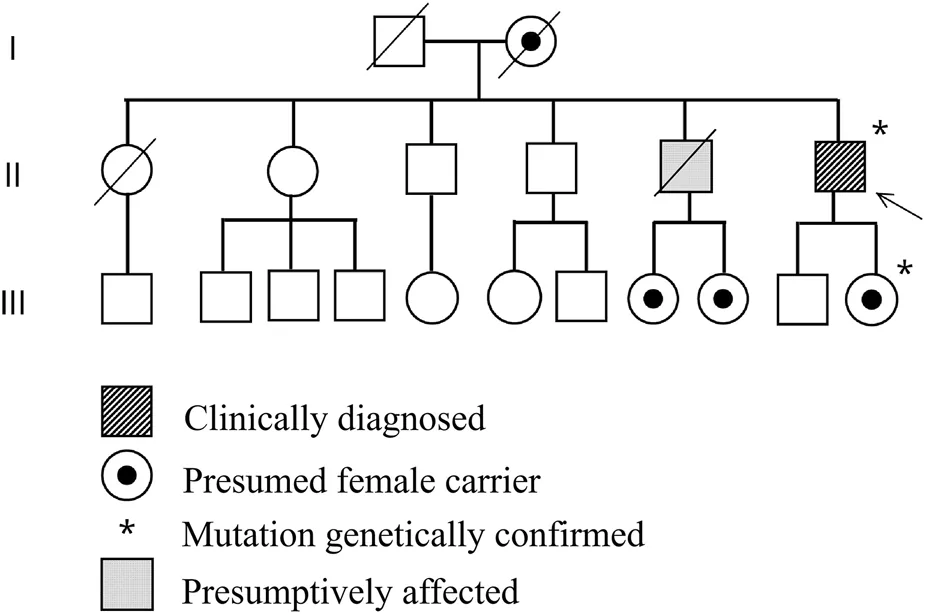
Pedigree of the Proband’s family. The arrow indicates the proband. Genetic testing was performed in the proband and his two children; his daughter was heterozygous for the variant. One younger brother reportedly died at age 50 after an approximately 10-year history of “muscle atrophy.” No other relatives were reported to have neuromuscular, psychiatric, or cardiac manifestations. This pedigree was created using Visual Paradigm Online (Visual Paradigm International Ltd., Hong Kong; accessed 12 December 2025).

### Physical and neurological examination

On general physical examination, his vital signs were stable and there were no remarkable cardiopulmonary or abdominal findings. On neurological examination, the patient showed impaired immediate memory and calculation ability. Formal cognitive screening demonstrated impairment, with scores of 17/30 on the Mini-Mental State Examination (MMSE) and 21/30 on the Montreal Cognitive Assessment (MoCA). Hyperkinetic movements were more evident when his attention was reduced. Cranial nerve examination was unremarkable and speech was relatively preserved. Flexion contractures were present at the interphalangeal joints of the left hand, and muscle atrophy was observed in the left upper limb and right hand. In the upper limbs, weakness was more prominent distally and on the left, involving elbow extension (left 3/5, right 4/5), wrist extension (left 2/5, right 3/5), wrist flexion (left 3/5, right 4+/5), finger extension (left 2/5, right 3/5), finger abduction (left 0/5, right 1/5), and grip strength (3/5 bilaterally), while shoulder abduction and elbow flexion were preserved (5/5). In the lower limbs, hip flexion was mildly weak bilaterally (4/5), whereas the other tested muscle groups were largely preserved (5/5). Muscle tone was reduced in all four limbs. Deep tendon reflexes were absent in all four limbs. No obvious pathological reflexes were noted. Vibratory sense was reduced in all four limbs, more pronounced distally and in the lower limbs, while pinprick sensation was intact. Rapid alternating movements of the upper limbs were slow bilaterally, while the heel-to-shin test was unsteady. Romberg sign was positive, with swaying in the standing position that worsened with eye closure. Gower sign was also positive. Gait assessment revealed intermittent deviation to either side and inability to walk in a straight line (Supplementary Video S1).

### Diagnostic assessment

The combination of progressive choreiform hyperkinetic movements, cognitive impairment, peripheral neuropathy, muscle weakness, and elevated creatine kinase (CK) raised suspicion for a core neuroacanthocytosis (NA) syndrome. Laboratory testing was unremarkable except for elevated aspartate aminotransferase (75 U/L, 15–40 U/L), alanine aminotransferase (133 U/L, 9–50 U/L), lactate dehydrogenase (396 U/L, 0–250 U/L), and hemoglobin A1c (8.1%, 4.5%–6.3%). Autoimmune, thyroid, ceruloplasmin, folate, and vitamin B12 tests were unrevealing. Cerebrospinal fluid examination was unremarkable. Cervical magnetic resonance imaging (MRI) showed disc protrusions with mild spinal cord compression, while brain MRI revealed bilateral caudate head atrophy (Figures 2A,B). Nerve conduction velocity (NCV) testing showed reduced compound muscle action potential (CMAP) and sensory nerve action potential (SNAP) amplitudes in multiple nerves with relatively mild conduction slowing, supporting a predominantly axonal sensorimotor polyneuropathy. Needle electromyography (EMG) showed spontaneous activity and prolonged motor unit duration without electrophysiological evidence of primary myopathy.

**FIGURE 2:**
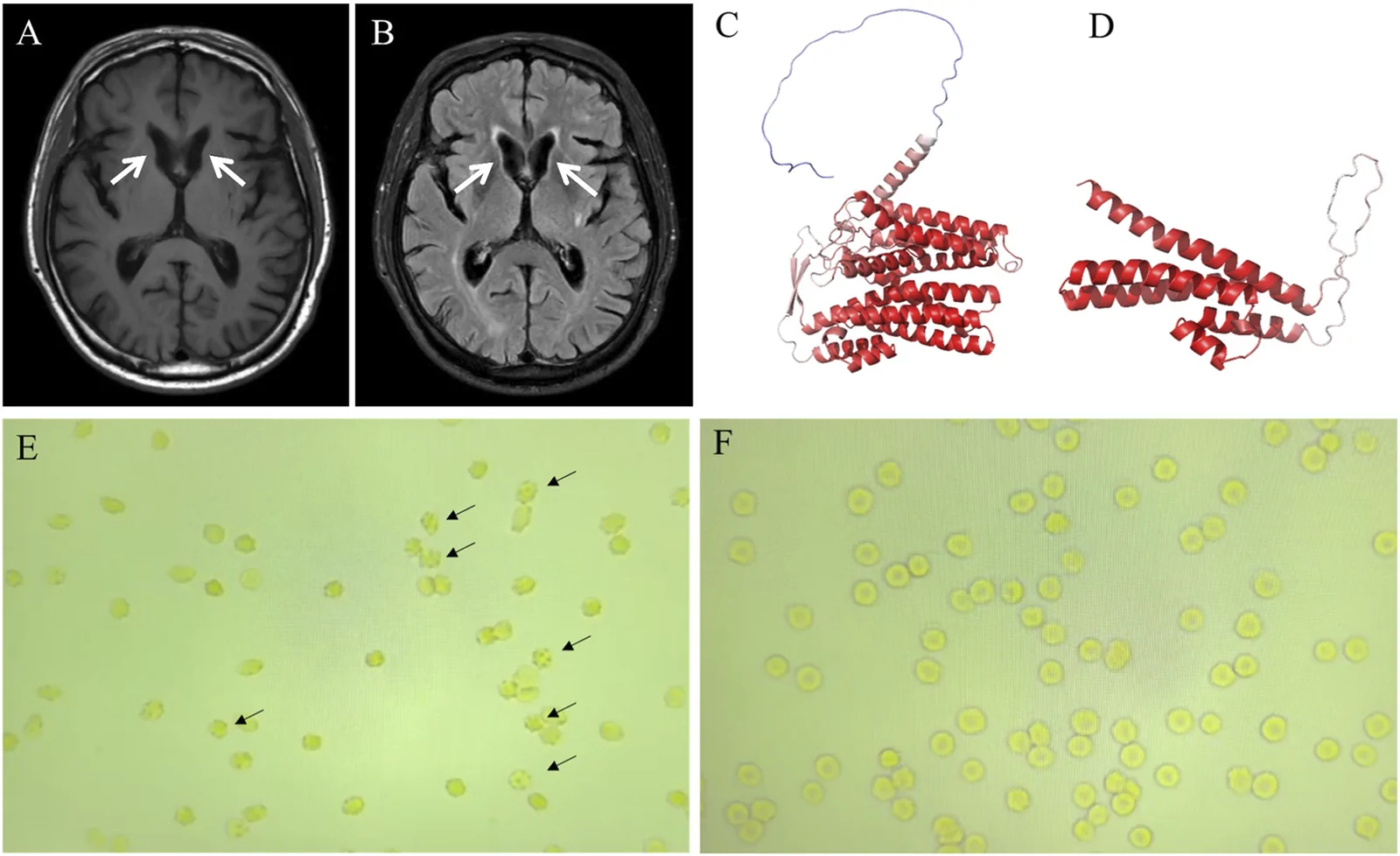
Neuroimaging, in silico modeling, and peripheral blood smear findings in the proband. **(A,B)** Axial brain MRI showed bilateral caudate head atrophy with enlargement of the frontal horns of the lateral ventricles on T1-weighted **(A)** and FLAIR **(B)** images. **(C,D)** ColabFold structural models were generated for the wild-type XK protein **(C)** and the truncated protein predicted from the *XK* p.Gln162Ter variant (residues 1-161) **(D)**. **(E,F)** Peripheral blood smears from the proband **(E)** and a healthy control **(F)**, prepared under identical conditions using EDTA-anticoagulated blood diluted 1:1 with isotonic saline, showed acanthocytosis in >60% of red blood cells in the proband (arrows) and no acanthocytes in the control.

More than 60% of red blood cells exhibited acanthocytosis on peripheral blood smears following 1:1 dilution with isotonic saline, whereas no acanthocytes were detected in the control (Figures 2E,F). Tube agglutination tests using anti-Kell (anti-K) and anti-Cellano (anti-k) antibodies showed markedly reduced reactivity. Whole-exome sequencing identified a hemizygous *XK* nonsense variant, NM_021083.4 (*XK*):c.484C > T, predicted to cause premature termination at codon 162, p.(Gln162Ter). Sanger sequencing confirmed the variant in the proband; consistent with X-linked transmission, his daughter was heterozygous, whereas his son did not carry the variant. In silico modeling predicted a marked C-terminal truncation with loss of multiple transmembrane helices (Figures 2C,D). Although the transcript was predicted to escape nonsense-mediated mRNA decay because the premature termination codon lay within 50 nt upstream of the final exon-exon junction, the truncated protein would lose most transmembrane domains and Cys347, which is essential for formation and surface expression of the Kell-XK complex on red blood cells (Jung et al., 2007; Popp and Maquat, 2013). For ACMG/AMP classification, given that loss of function is an established disease mechanism for *XK*-related McLeod syndrome, the nonsense variant fulfilled PVS1; its absence from gnomAD (AC = 0, no hemizygotes) supported PM2; and the highly specific phenotype supported PP4, which together supported a pathogenic classification (Richards et al., 2015). The reduced anti-K and anti-k reactivity provided additional serological evidence supporting the McLeod phenotype and could be considered as PS3_Supporting under ClinGen SVI criteria (Brnich et al., 2020). Together, these findings supported the diagnosis of McLeod syndrome.

Since cardiomyopathy and arrhythmias are common complications of MLS, cardiac evaluation was performed despite the absence of cardiopulmonary symptoms. Although CK-MB mass (24.5 μg/L, ≤5 μg/L) and myoglobin (838 μg/L, ≤110 μg/L) were elevated, hs-cTnI and NT-proBNP were within normal ranges, suggesting that the elevated CK-MB and myoglobin reflected skeletal rather than cardiac muscle injury. Echocardiography demonstrated mildly increased left ventricular (LV) wall thickness, grade II diastolic dysfunction with preserved left ventricular ejection fraction (61%), and mild dilatation of the ascending aorta and the pulmonary trunk. A 24-h Holter electrocardiogram (ECG) showed fewer than 100 premature atrial or ventricular contractions, with no episodes of non-sustained ventricular tachycardia.

### Therapeutic interventions and disease management

Tiapride or deutetrabenazine was recommended for symptomatic control of hyperkinetic movements. As the patient’s cardiac involvement was considered subclinical, cardiac magnetic resonance (CMR) was suggested for further myocardial assessment, along with regular cardiology follow-up. Given the proband’s immunohematologic findings, avoidance of nonessential transfusions was advised; if transfusion became necessary, early coordination with transfusion services to identify compatible units and consideration of autologous blood banking when feasible were recommended. A timeline of the proband’s episodes of care is provided in Table 1.

**TABLE 1:** Timeline of the Proband’s episodes of care.

Date	Episodes of care	Key clinical features
2017	Symptom onset	Unsteady gait; hyperkinetic movements
2023	First medical evaluation	Cognitive decline; progressive clumsiness; distal- predominant weakness; head CT (−)
2025-03	Second medical evaluation	Progressive clumsiness and distal-predominant weakness; right-limb numbness; CK: 5537 U/L; brain MRI: caudate atrophy; NCV/EMG: peripheral neurogenic findings; WES: *XK* c.484C > T; No benefit from mecobalamin treatment
2025-08	Third medical evaluation (outpatient, our center)	Neurological exam: hyperkinetic movements, distal- predominant weakness and atrophy, generalized areflexia; CK: 3655 U/L; inpatient admission
2025-11	Inpatient diagnostic workup at our center	Blood smear: acanthocytosis; tube agglutination tests (anti-K/anti-k): markedly reduced reactivity; diagnosed with MLS; tiapride for symptomatic control; cardiac evaluation, transfusion planning and genetic counseling

Abbreviations: CT, computed tomography; CK, creatine kinase; MRI, magnetic resonance imaging; NCV, nerve conduction velocity; EMG, electromyography; WES, whole-exome sequencing; anti-K, anti-Kell antibody; anti-k, anti-Cellano antibody; MLS, McLeod syndrome.

### Follow-up and outcomes

At telephone follow-up after discharge, the patient continued to have hyperkinetic movements, but tiapride or deutetrabenazine had not been initiated at the time of writing because of concerns about adverse effects. Family testing confirmed that his daughter had similarly reduced reactivity to anti-Kell (anti-K) and anti-Cellano (anti-k). She was advised to take transfusion precautions, and prenatal genetic counseling was suggested if pregnancy was planned. Baseline and longitudinal cardiac surveillance for arrhythmias and cardiomyopathy was also recommended for her.

### Patient perspective

The patient and his family felt relieved to receive a definitive diagnosis after years of progressive symptoms. They understood the need for cardiac surveillance, transfusion precautions, and genetic counseling. The patient expressed hope that further research into disease mechanisms could lead to targeted treatments and stated his willingness to participate in future clinical research if available.

## Discussion

This report describes a mainland Chinese Han family with McLeod syndrome caused by the *XK* nonsense variant c.484C > T, resulting in a premature stop codon at position 162. To our knowledge, this variant has not previously been reported in association with McLeod syndrome in Asian populations and was not listed in the ISBT blood group database at the time of writing (Walker et al., 2024; ISBT, 2021). Based on our literature review, we identified only one previously reported patient of African ancestry with McLeod syndrome carrying the *XK* c.484C > T variant (Walker et al., 2024). Nevertheless, no consistent genotype-phenotype correlation has been established in MLS, and expressivity can vary substantially even among individuals carrying the same *XK* variant (Jung et al., 2011; Walker et al., 2024; Braun and Jung, 2024; Díaz-Manera et al., 2018; Walker, 2015). Along the same lines, reports of Chinese cases have also demonstrated marked clinical and genetic heterogeneity: two affected brothers from one family, both carrying the *XK* variant c.856_860delCTCTA, differed substantially in movement disorder severity, neuromuscular involvement, CK levels, seizures, and disease progression, whereas a recent series of three Chinese MLS probands also identified variable clinical courses (Man et al., 2014; Yu et al., 2024).

In our proband, poorly controlled diabetes and cervical degenerative changes may have contributed to individual symptoms but did not explain the overall combination of central and peripheral neurologic involvement, generalized areflexia, and elevated CK and aminotransferase levels, prompting consideration of a rarer disorder such as a core neuroacanthocytosis syndrome (Jung et al., 2004; Walker et al., 2024). Although the detection of acanthocytes is not required for diagnosis, peripheral blood smear examination remains a helpful screening step, and wet preparations after 1:1 dilution of EDTA-anticoagulated blood with isotonic saline may improve detection sensitivity (Jung et al., 2011; Danek et al., 2001; Storch et al., 2005). Because XK and VPS13A disease overlap clinically, definitive differentiation of MLS from VPS13A disease requires identification of a pathogenic *XK* variant and/or demonstration of the McLeod red cell phenotype (Jung et al., 2007; Jung et al., 2004; Walker et al., 2024). Ideally, direct Kx antigen testing should be performed to confirm the McLeod red cell phenotype, while a more extensive Kell antigen panel would allow more comprehensive immunohematologic characterization (Jung et al., 2007; Jung et al., 2004). However, direct Kx testing and extended Kell phenotyping could not be performed during the diagnostic work-up because the required serologic reagents were unavailable at our institution. Despite these limitations, the overall clinical presentation together with marked acanthocytosis was suggestive of a core neuroacanthocytosis syndrome. The hemizygous pathogenic *XK* variant established the diagnosis of MLS, while markedly reduced reactivity with anti-Kell (anti-K) and anti-Cellano (anti-k) provided supportive immunohematologic evidence.

Notably, the patient’s impaired vibration sense with preserved pinprick sensation, together with generalized areflexia and reduced SNAP amplitudes, suggested predominant large-fiber sensory involvement with relative sparing of small-fiber modalities, consistent with reported axonal neuropathy and large myelinated fiber involvement in MLS (Jung et al., 2004; Danek et al., 2001). In addition, the positive Gower sign and marked hyperCKemia raised the possibility of concomitant skeletal muscle involvement. However, needle EMG showed predominantly neurogenic changes without clear evidence of primary myopathy. Further evaluation with muscle MRI and muscle biopsy was recommended for clarification, but was not pursued because the patient declined further evaluation.

Mechanistically, accumulating evidence suggests that MLS (caused by XK dysfunction) and ChAc (caused by VPS13A dysfunction) converge on a shared membrane lipid-homeostasis pathway centered on the VPS13A-XK complex. VPS13A, an ER-anchored bridge-like lipid transfer protein, can be recruited to plasma membrane contact sites through binding to XK, a lipid scramblase, thereby coupling lipid transfer with phospholipid equilibration across the plasma membrane bilayer (Guillén-Samander et al., 2022; Park and Neiman, 2020; Park et al., 2022; Amos et al., 2023). Pathogenic variants in either gene may disrupt membrane stability maintained by this complex, providing a molecular basis for the overlapping phenotypes of the two disorders, whereas their distinct features may reflect the specific functional consequences of loss of XK or VPS13A. These observations suggest that excitable cells with high demands for plasma membrane phospholipid asymmetry and homeostasis—particularly striatal neurons, long peripheral axons, and skeletal and cardiac muscle—may be especially vulnerable to loss of XK function; however, the basis for this tissue selectivity remains to be elucidated.

Management of MLS remains supportive and anticipatory, including symptomatic treatment of hyperkinetic movement disorders, transfusion precautions with early coordination to secure compatible blood products, and genetic counseling (Jung et al., 2004; Walker, 2015). Given that cardiomyopathy and arrhythmias contribute substantially to morbidity and mortality in MLS, including sudden cardiac death, vigilant longitudinal cardiac surveillance is essential. Accordingly, cardiac magnetic resonance (CMR) imaging may aid in the early detection of myocardial fibrosis, and implantation of an implantable cardioverter-defibrillator (ICD) should be considered when clinically indicated (Braun and Jung, 2024; Reuss et al., 2025; Oechslin et al., 2009; Boppana et al., 2023). Female carriers are generally asymptomatic, although isolated cases have been reported with hyperkinetic movements, cognitive impairment, polyneuropathy, myopathic and neurogenic changes on EMG, elevated CK, and acanthocytosis, sometimes in association with skewed X-chromosome inactivation (Jung et al., 2004; Braun and Jung, 2024). Although the frequency and spectrum of manifestations in female carriers, particularly cardiac involvement, remain poorly characterized, the recognized potential for female carriers to develop clinical manifestations, together with the potentially serious cardiac complications of MLS, supports their longitudinal cardiac assessment, along with neurologic evaluation, transfusion precautions and reproductive genetic counseling.

## Conclusion

McLeod syndrome should be considered in men presenting with adult-onset hyperkinetic movements, upper-limb distal-predominant weakness with mild proximal lower-limb weakness, generalized areflexia, and hyperCKemia, especially when acanthocytosis is identified. Based on the available literature and blood group database review, our proband and family appear to represent one of the first reported East Asian families with McLeod syndrome associated with the *XK* nonsense variant c.484C > T, which was not listed in the ISBT *XK* allele table at the time of writing. Recognition of such cases may facilitate accurate diagnosis through blood group phenotyping and *XK* sequencing, and support timely cardiac surveillance, transfusion planning, genetic counseling, and symptomatic management.

## Data Availability

The original contributions presented in the study are included in the article/Supplementary Material, further inquiries can be directed to the corresponding author.

## Supplementary material

SUPPLEMENTARY VIDEO S1 Hyperkinetic Movements and Gait Instability in the Proband. While seated, the patient exhibits hyperkinetic movements, including tic-like scratching and tugging at his clothing, accompanied by intermittent sighing. The subsequent walking segment demonstrates gait instability, with an inability to walk in a straight line.
